# Understanding Platelets in Infectious and Allergic Lung Diseases

**DOI:** 10.3390/ijms20071730

**Published:** 2019-04-08

**Authors:** Cristina Gomez-Casado, Alma Villaseñor, Alba Rodriguez-Nogales, Jose Luis Bueno, Domingo Barber, Maria M. Escribese

**Affiliations:** 1 Institute of Applied Molecular Medicine, Department of Basic Medical Sciences, Faculty of Medicine, San Pablo CEU University, 28668 Madrid, Spain; alma.villasenor@ceu.es (A.V.); domingo.barberhernandez@ceu.es (D.B.); mariamarta.escribesealonso@ceu.es (M.M.E.); 2 Research Laboratory, Allergy Unit, University Hospital of Malaga-IBIMA, 29010 Malaga, Spain; albarnogales@gmail.com; 3 Blood Transfusion & Non-Transfusional Hemotherapy Unit, University Hospital Puerta de Hierro-Majadahonda, 28220 Madrid, Spain; jolubuca1898@gmail.com

**Keywords:** platelets, mediators, allergic inflammation, infectious disease

## Abstract

Emerging evidence suggests that platelets, cytoplasmic fragments derived from megakaryocytes, can no longer be considered just as mediators in hemostasis and coagulation processes, but as key modulators of immunity. Platelets have received increasing attention as the emergence of new methodologies has allowed the characterization of their components and functions in the immune continuum. Platelet activation in infectious and allergic lung diseases has been well documented and associated with bacterial infections reproduced in several animal models of pulmonary bacterial infections. Direct interactions between platelets and bacteria have been associated with increased pulmonary platelet accumulation, whereas bacterial-derived toxins have also been reported to modulate platelet function. Recently, platelets have been found extravascular in the lungs of patients with asthma, and in animal models of allergic lung inflammation. Their ability to interact with immune and endothelial cells and secrete immune mediators makes them one attractive target for biomarker identification that will help characterize their contribution to lung diseases. Here, we present an original review of the last advances in the platelet field with a focus on the contribution of platelets to respiratory infections and allergic-mediated diseases.

## 1. Introduction

Platelets are anucleate cell fragments derived from megakaryocytes that have been considered as mere “sacks” of mediators specialized in hemostasis and coagulation [1]. Recently, platelets have gained increasing importance in the field of inflammation and immunology [1]. New methodologies have allowed obtaining purified populations of non-activated platelets that facilitate the characterization of their components. Moreover, recent reports demonstrate their ability to release mediators that can be recognized by other cell types, such as endothelial cells, macrophages, T lymphocytes, neutrophils, and mast cells, showing their potential role in pathologies such as allergy or infectious lung diseases. Altogether, the current knowledge of platelet biology indicates that they can no longer be considered as “simple” cell fragments.

Here, we present an original review of the last advances in platelet field with a focus on allergic and infectious lung diseases.

## 2. Platelet Biology

Platelets are generated from megakaryocytes in a multi-step process called thrombopoiesis regulated by thrombopoietin [2]. Thrombopoietin stimulates its receptor in megakaryocytes to induce the genesis of pro-platelets via a mechanism activated by low platelet counts. Platelet counts in blood are controlled by the rates of production and removal, involving mechanisms of platelet clearance, activation or ageing [3]. 

Platelets are the most numerous circulating cell type (≈200,000/µL blood in humans) with an immune function [4]. They circulate for 7–10 days and undergo programmed cell death through apoptosis regulated by the antagonistic balance between Bcl-x(L) and Bak. This constitutes a molecular clock that determines platelet lifespan [5]. Activated or apoptotic platelets expose phosphatidylserine on their outer membrane surface and are cleared via scavenging receptors on phagocytic cells in the liver and other organs [6].

### 2.1. Platelets as Coagulation Factors

The traditionally attributed role of platelets is to assure hemostasis. Platelets become rapidly activated and adhere tightly to other platelets and to the wall vessel as soon as damage is found. Briefly, platelets bind to von Willebrand factor (vWF), which forms a bridge with exposed collagen on the injury and glycoprotein Ib (GPIb)/V/IX receptor complex on the platelet membrane. The exposed collagen also binds directly to platelet GPIa/IIa and GPVI receptors to induce platelet activation in a positive feedback loop. Platelets then release mediators such as ADP and serotonin that activate platelet G protein-coupled receptors. This process results in increased levels of cytosolic calcium and activates signaling pathways leading to platelet shape change and activation of integrins, enhancing the adhesion of platelets to the endothelial wall. Furthermore, ADP acts on platelet P2Y1 and P2Y12 G-protein-coupled receptors to sustain platelet activation [7,8]. Finally, the activation of the GPIIb/IIIa receptor results in the cross-linking of fibrinogen or vWF with their receptors (integrin aIIbβ3) leading to platelet aggregation. This promotes the recruitment of additional platelets to the site of vascular injury allowing the formation of the thrombus [9,10].

Platelets also participate in biological processes such as vascular integrity, tissue regeneration and angiogenesis, and lymphatic vessel development [11,12,13,14,15,16,17,18]. 

### 2.2. Platelets as Immune Mediators

Platelets count with several organelles such as mitochondria, lysosomes and peroxisomes, and a plethora of intracellular immune mediators stored in granules and vesicles [19]. Moreover, platelets express a high number of membrane receptors and contain cytoplasmic mRNA, which can synthetize a limited number of proteins and miRNA [4,9]. These receptors and proteins allow them to interact with leukocytes and endothelial cells, both by contact-dependent mechanisms and through secreted immune mediators. Thus, platelets can modulate immune responses at the sites of platelet activation systemically [20].

Platelet receptors and the molecules stored in platelet granules govern platelet functions. These molecules are listed in Table 1. There are three types of platelet granules: α-granules, dense granules and lysosomal granules. Additionally, a potential new type of granule termed a T-granule has been described [21]. α-granules are the most numerous (50–60 per platelet) and largest (200–400 nm) granules. They contain a large variety of proteins, close to 300, including a diverse range of chemokines, such as CXCL1, platelet factor 4 (PF4), CXCL5, CXCL7, CXCL12, macrophage inflammatory protein (MIP)-1α and regulated on activation normal T expressed and secreted (RANTES) [22]. Dense granules are smaller (~150 nm), less abundant (3–8 per platelet) and store small molecules, such as ADP, ATP, inorganic polyphosphate, pyrophosphate, histamine, serotonin, and calcium. Finally, lysosomal granules are sparse and contain proteases and glycosidases [10,23]. Upon platelet stimulation, granules undergo regulated exocytosis and release their content into the extracellular environment. In addition, molecules found on the inner granule membrane become surface-expressed. Many of these granule-derived molecules are immune mediators. 

This is the case of P-selectin, which is one of the most bioactive molecules contained in α-granules and involved in inflammation. It promotes platelet aggregation and platelet-endothelial and platelet-leukocyte interactions [24]. Other α-granule constituents such as PF4 or RANTES are immune mediators that recruit and activate immune cells or induce endothelial cell inflammation [25,26]. The inflammatory roles of most α-granule-derived chemokines, cytokines and adhesion molecules are well described. However, the direct contribution of dense granule constituents to immune responses is still largely unexplored. It is nevertheless known that serotonin, contained in dense granules, increases monocyte differentiation into dendritic cells (DCs) [27] and early naïve T-cell activation [28]. Moreover, platelets can recruit and activate DCs via integrin alphaMbeta2 (Mac-1) [29]. In addition, DC expression of T-cell co-stimulatory molecules CD80 and CD86 is increased by activated platelets in a contact-independent manner leading to a stronger and more rapid T-cell response [30]. In turn, T cells may activate platelets through a T-cell CD40L/platelet CD40 interaction leading to platelet release of RANTES and further T-cell recruitment [31]. Platelets are also the major source of soluble CD40L, which induces B-cell production of immunoglobulin (Ig)G, by activating DCs and promoting B-cell isotype switching [24]. It has also been suggested that platelets enhance signals needed for adaptive humoral immunity and germinal center formation [32].

Activated platelets also release interleukin-1β (IL-1β), which is not granule-stored but produced upon platelet stimulation [33]. Typical markers of platelet activation (granule exocytosis and integrin expression) are increased rapidly after platelet stimulation (seconds to minutes), but the release of IL-1β from stimulated platelets occurs over hours [34].

Platelets affect all phases of immune responses. Their interactions with immune cells likely mediate beneficial outcomes in limiting infection and maintaining hemostasis [35]. However, continued platelet interactions with leukocytes or endothelial cells can also lead to adverse effects from excessive immune stimulation and inflammatory insult [4].

### 2.3. Metabolic Plasticity of Platelets

Despite being small and lacking a nucleus, platelets possess extraordinary metabolic machinery. The metabolism of platelets is not fully known yet; however, it has been estimated that at their basal metabolic state, ATP is generated by both mitochondrial oxidative phosphorylation (OXPHOS) (35%) and glycolysis (65%) [36]. During platelet activation, fatty acid oxidation and glutaminolysis is promoted to meet the energetic needs for aggregation [36]. Fatty acids and L-glutamine are required for OXPHOS, and if necessary, platelets can uptake extracellular fatty acids [36]. Interestingly, platelets proved to have metabolic plasticity, which allows them to compensate the energetic demand driven by activation and aggregation switching from one to another metabolic route [36]. These two important ATP-generating pathways have become relevant in translational research, where it has been observed that severe phenotypes of allergic and respiratory diseases show a bioenergetic dysfunction in platelets and leukocytes [37]. As an example of dysfunction, decreased glycolytic function in platelets from asthmatic patients has shown to be compensated by increased tricarboxylic acid (TCA) cycle activity leading to a re-direction of their metabolism towards mitochondrial metabolism, which might increase oxidative injury in asthma [38]. 

## 3. Platelets in Respiratory Allergic Inflammation

Platelet role in allergic diseases has been extensively reviewed by Page C and Pitchford S [39]. Platelet abnormalities in patients with allergy have been reported in the literature for more than 50 years [40]. Changes in the mean platelet volume (MPV) and platelet mass have been described in atopic subjects, which may correlate with changes in platelet function or activation [41,42]. Furthermore, a reduction in the platelet lifespan has been observed in asthmatic patients, which suggests that there is continuous platelet consumption because of chronic activation [43]. However, there is contradictory information, since other groups have reported no differences in platelet lifespan between healthy individuals and patients with asthma [44,45]. This suggests that the mechanisms governing platelet production, homing and lifespan in inflammatory states are complex. 

Additionally, several studies long ago reported that platelets isolated from the peripheral blood of allergic patients usually presented reduced responsiveness to aggregatory stimuli ex vivo, and limited storage of immune mediators, suggesting that they may behave in an “exhausted” manner [46,47,48,49].

### 3.1. Alterations in Platelet Functions

Recently, Obeso et al. described altered platelet functions in a multi-omics analysis of severe respiratory allergic phenotype [37]. Significant transcripts associated with platelet functions, protein synthesis, histone modification, and fatty acid metabolism were downregulated in the severe allergic group. Moreover, a complete metabolic fingerprint of these patients revealed significant differences in energy metabolism, sphingolipids, fatty acids and lysophospholipids. A decrease in carbohydrates, carnitine and pyruvate together with an increase in lactate suggested an aerobic glycolysis metabolism in severe allergic patients. In agreement with these results, existing omics-based studies have reported that immune cells change their metabolism to fulfill the increasing demand resulting from the need to synthetize biological precursors. This is known as Warburg metabolism (or aerobic glycolysis) [50,51] and has been described in tumors, asthma, and inflammation [52,53,54]. Furthermore, other metabolites including lysophospholipids, sphingosine-1-phosphate (S1P), sphinganine-1-phosphate and lauric, myristic, palmitic and oleic fatty acids were augmented in the severe group. The increase in arachidonic acid precursors—palmitic and oleic acids, along with shorter fatty acids: myristic and lauric acids—was observed in severe allergic patients. The transcellular metabolism of arachidonic acid allows platelets to enhance the formation and conversion of leucocyte-derived leukotrienes (LT)C4, LTD4 and LTE4 [55,56]. Moreover, platelets are one of the main sources of S1P. This metabolite, which is a key signal molecule inducing airway smooth muscle hyper-reactivity and lung inflammation, was increased in the severe group [57]. Furthermore, it can bind to the S1P receptors on the mast cell surface producing the degranulation of these cells [58]. This fact is crucial in the allergic inflammatory process. To sum up, the downregulated platelet transcripts and the increase in plasma S1P levels points to an altered platelet functionality in the severe phenotype.

### 3.2. Platelet Contribution in Lung Allergic Sensitization and Inflammation

Additionally, platelets are likely to influence lung regeneration and inappropriate airway remodeling after injury, since the depletion of platelets in murine models of chronic allergic inflammation led to a comprehensive suppression of remodeling features such as smooth muscle hyperplasia, sub-epithelial fibrosis, collagen deposition, or epithelial hyperplasia [59]. However, the interplay between platelets and mucosal cells is extremely complex and involves a plethora of mediators at various levels. In nasal polyposis, a comorbidity that shares histological and biochemical features of allergy and inflammation, platelet contribution has been already reported. Eosinophilic infiltrate is the most characteristic feature of nasal polyps, particularly in patients with aspirin-sensitive asthma (ASA), which is considered as a severe phenotype of this pathology [60,61]. Platelet-activating factor (PAF) is a phospholipid-derived mediator, which acts as a potent activator of human eosinophils. Several studies have reported PAF activity in nasal polyps of ASA patients and patients with chronic sinusitis [60]. Furthermore, PAF is capable of mimicking many aspects of the allergic condition such as the induction of leukocyte-dependent histamine release from platelets [62]. Moreover, PAF affects skin barrier integrity [63] and activates epithelial cells to release alarmins, such as IL-33. In fact, both platelets and megakaryocytes constitutively express IL-33 and release it when activated [64].

Furthermore, increasing evidence links PAF to the pathology of anaphylaxis in animal models [65,66] and humans, where circulating PAF levels have been correlated with the severity of anaphylactic reactions [67]. Arias K. et al., have reported that the severity of anaphylactic reactions caused by nuts could be substantially reduced by blocking PAF signaling in mice [68]. Moreover, the blockade of PAF receptor was associated with decreased vascular leakage and a reduction in histamine and leukotriene levels in plasma. 

IgE-mediated anaphylaxis is thought to be the main anaphylactic pathway. However, increasing evidence from animal models supports the existence of an IgG-dependent alternative pathway. The main mediator released in the alternative pathway is PAF instead of histamine [69]. In this sense, Jimenez-Saiz et al. [70] recently reported that IgG1+ B-cell immunity prevailed in the initial stages of epicutaneous sensitization to nut allergens. Accordingly, clinical reactivity at this stage was driven by the alternative pathway, involving PAF [69,71]. 

Recently, it has also been reported that platelets play a central role in allergic sensitization [72]. In this study, Amison et al. investigated the role of platelets at the time of allergen sensitization in a mouse model of allergy to ovalbumin (OVA) through platelet depletion experiments restricted to the period of sensitization. They sensitized the mice to OVA and subsequently exposed them to aerosolized allergen (OVA challenge), analyzed lung CD11c+ (a marker for antigen-presenting cells-APCs-) activation, co-localization with platelets, and some other indices of inflammation. They observed that platelets co-localized with airway CD11c+ cells, and this association increased after allergen sensitization as well as after subsequent allergen exposure. Temporary platelet depletion (>95%) at the time of allergen sensitization led to a suppression of IgE and IL-4 synthesis and to a decrease in the pulmonary recruitment of eosinophils, macrophages, and lymphocytes after subsequent allergen exposure. Moreover, pulmonary CD11c+ cell recruitment was suppressed in these mice after the allergen challenge, suggesting that the migration of CD11c+ cells in vivo may be dependent on direct platelet recognition of the allergen. Thus, according to these findings, platelets are necessary for efficient sensitization to allergens. This propagates the subsequent inflammatory response during secondary allergen exposure and increases platelet association with airway CD11c+ cells.

Besides, platelets express an array of receptors that may be relevant in asthma and allergic airway inflammation. These include chemokine receptors (CCR1, CCR3, CCR4, and CXCR4 receptors) [73], Ig receptors (FcγRI, FcγRII, FcγRIII; FcεRI, FcεRII, FcαRI/CD89) [74], TLRs (TLR2, TLR4, and TLR9) [75] and certain adhesion molecules (PSGL-1, P-Selectin and ICAM-2) [76,77]. 

The function of the high affinity IgE receptor (FcεRI) on platelets is not well characterized. Recently, FcεRIα has been identified as the ligand for PEAR1 (Platelet Endothelium Activation Receptor 1, also known as multiple epidermal growth factor-like domain protein 12 (MEGF12) or JEDI-1), a platelet cell surface receptor that was originally identified as a protein phosphorylated in response to platelet aggregation [78].

In healthy individuals, the concentration of circulating IgE is very low at 0.5 nM [79]. However, when circulating levels of IgE are increased, e.g., in atopic patients, the amount of IgE-free FcεRI on the platelet surface is significantly decreased. Thus, less FcεRI is available for PEAR1 binding [79]. This is consistent with reports of a systemic lack of platelet responsiveness in atopic individuals, an observation that was correlated with elevated IgE levels [46,49,80,81]. Others, however, have not replicated these findings [82,83], suggesting that there is a more complex relationship between circulating IgE levels and platelet functions. In humans, a humanized anti-IgE monoclonal antibody (omalizumab) is currently licensed for the treatment of severe allergic asthma by reducing circulating IgE levels by 99% and down-regulating FcεRI on mast cells and basophils [84,85,86]. Thus, omalizumab treatment could lead to alterations in the regulation of PEAR1 signaling. Indeed, concerns have recently been raised about an increased risk of arterial thrombotic events linked to the use of omalizumab [87]. Transient decreases in peripheral blood platelet counts have also been reported in humans treated with omalizumab. However, clinical data found no risk of thrombocytopenia for these patients [88].

### 3.3. Lung Platelets

A recent study in mice has revealed that platelets are generated in the lung from resident megakaryocytes [89]. According to this study, more than 10 million platelets are produced per hour in the lungs [89]. This means that the lung is responsible for approximately 50% of total platelet production.

Previous studies demonstrated that blood leaving the lungs contained more platelets and fewer megakaryocytes than blood entering the lungs [90,91]. Interactions between megakaryocytes and endothelial cells through GPIb–vWF signaling promote pro-platelet formation in vitro [92]. Considering that vWF levels are particularly high in the pulmonary arteries [93], this pathway could regulate platelet production from lung megakaryocytes. Lefrançais et al. demonstrated that in addition to intravascular megakaryocytes, these cells were also found in the perivascular lung interstitium. The relative proportions of these populations are 15% intravascular and 85% extravascular. Furthermore, only intravascular megakaryocytes of extra-pulmonary origin released platelets. However, platelets found in the pulmonary arteries could present a characteristic phenotype influenced by the local milieu, which may confer them with the ability to modulate inflammatory or fibrotic lung diseases. This highlights the importance of understanding lungs as bioreactors to produce mature platelets from megakaryocytes, which could open new research lines to improve therapeutic approaches in lung diseases. 

In conclusion, we are now beginning to understand platelet contribution to allergic sensitization and inflammation. It seems that there is a dichotomy in platelet activation during inflammation compared to hemostasis, since patients with allergic diseases have a mild hemostatic defect despite significant platelet activation. This suggests that platelet activation mechanisms might be exploited for developing novel anti-inflammatory therapies without affecting the function of platelets in hemostasis.

## 4. Platelets in Infectious Diseases

Platelets have been associated with inflammatory and immune complications of malaria, sepsis, human immunodeficiency virus (HIV) and influenza, all of them causing a large public health burden worldwide [4]. Platelets express TLRs on their surface [94] that recognize, among others, bacterial peptidoglycans (TLR2) and LPS (TLR4) in Gram-positive and Gram-negative bacteria [95,96,97,98]. Indeed, platelet shape change, platelet adhesion, formation of platelet–leukocyte complexes, elevated P-selectin expression, and platelet granule release have all been detected after exposure to bacterial products or by directly interacting with bacterial pathogens such as *Staphylococcus aureus*, *Streptococcus pyogenes*, *Escherichia coli*, and *Clostridium perfringens* [99,100,101].

### 4.1. Platelets in Bacterial Infection

Lower respiratory infections are the most deadly communicable disease worldwide [102], of which *Streptococcus pneumoniae* (or pneumococcus) infection is the most common [103]. Recent studies indicate that several deaths from pneumonia seem to be related to cardiovascular events occurring during the infection, and there also seems to be an important emerging role for platelet activation underlying some of these events [104]. Activation of platelets by pneumococcus in vitro was already reported [105]. More recently, Tunjungputri et al. associated the expression of the pblB gene by pneumococcus with significantly increased platelet activation and 30-day mortality [106]. There are still few clinical studies on the platelet role in pneumococcal infection. The inclusion of platelet biomarkers with predictive potential is of high importance given that about one-third of the patients hospitalized for pneumonia experience intra-hospital cardiovascular events, including heart failure, myocardial infarction, ischemic stroke and venous thrombosis [107]. A combined measurement of certain platelet CXC chemokines, platelet factor-4 (PF4) and β-thromboglobulin could be useful for characterizing these patients [108,109]. Additionally, miR-126, a microRNA, has been recently reported as a systemic biomarker of platelet activation [110,111]. More information on platelet biomarkers related to pneumococcal infections can be found in the extensive review by Anderson R and Feldman C [112]. In addition to pneumococcal infection, platelet activation associated with *Staphylococcus aureus*- and *Klebsiella pneumoniae*-induced sepsis has been described in several animal models of pulmonary bacterial infections [113]. In these models, thrombocytopenia was associated with higher pulmonary bacterial load and increased systemic infection [114,115]. In humans, increases in the expression of platelet activation markers in patients hospitalized with numerous infectious diseases have been reported. These markers include increased levels of surface P-selectin expression, increased granular secretion [116] and increases in the numbers of platelet–monocyte and platelet–neutrophil complexes in the circulation. Patients with sepsis also usually present thrombocytopenia, which has been associated with a worse prognosis and increased mortality when compared with patients with normal platelet levels [117]. Moreover, platelet-interactions with other bacterial pathogens such as *S. aureus, Helicobacter pylori* and *Pseudomonas aeruginosa* have also been reported both in vitro and in vivo. An increased platelet accumulation in the lungs was associated to these interactions with bacterial pathogens [118]. Direct interactions between platelets and bacteria lead to subsequent engulfment of *S. aureus*, *S. pneumoniae*, and *P. aeruginosa* by platelets in order to remove bacteria from the infected tissue [113]. There is evidence that platelets play an important role in the host response to pulmonary bacterial infection with *P. aeruginosa*. It has been demonstrated that experimentally-induced platelet depletion allows increased pulmonary bacterial growth and systemic bacterial dissemination. Thus, platelets play a significant role in restraining bacterial infections to the lung [119]. Moreover, platelets can also increase the antimicrobial effects of other immune cells [120,121]. In turn, bacterial-derived toxins can also modulate platelet function [101]. Toxin-platelet interactions can result in either platelet activation or inhibition, depending on the bacteria and bacterial toxin. The nature of the platelet response may be highly relevant to disease pathogenesis.

### 4.2. Platelets in Viral Infection

Besides bacteria, platelets can internalize HIV and lentivirus through the receptors C-type lectin-like receptor (CLEC)-2 and DC-Specific Intercellular adhesion molecule-3-Grabbing Non-integrin (DC-SIGN) expressed on platelet membrane [113,122]. Like HIV infection, platelet-monocyte aggregates are elevated in influenza infection [123]. Rondina et al. found that thrombocytopenia and leukopenia are common during influenza infection, as previously reported for other viral respiratory infections [124]. They observed in vivo platelet activation in these patients. Although platelet contribution to influenza infection is still largely unexplored, there is evidence that human and mouse platelets have a receptor for influenza viruses [125].

Respiratory syncytial virus (RSV) is a major cause of lower respiratory tract infections in young children and immune compromised patients [126]. The interaction of platelets–leukocytes and the subsequent alteration in cytokine production may be one of the mechanisms involved in host defense against RSV [127]. Likewise, platelets can reduce RSV infection of monocytes and monocyte activation by enhancing IFN production from leukocytes and by internalizing viral particles [128]. Moreover, analysis of the gene expression profiles from infants with lower respiratory tract infections of different viral etiologies showed that the overexpression of platelet-related genes is specific for RSV [129]. Thus, platelet-mediated reduction of monocyte activation during RSV infection may be important for preventing lung inflammation.

**Figure 1:**
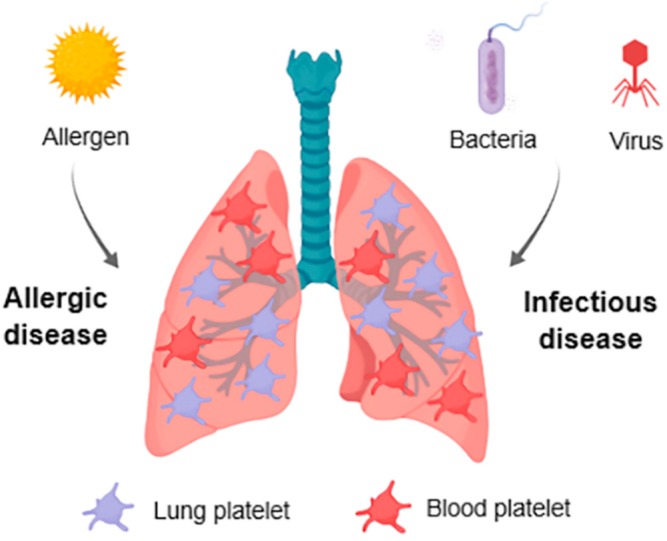
Graphical scheme summarizing platelet involvement in lung diseases. Platelets in the lungs interact with bacteria (and bacterial-derived toxins), viruses and aeroallergens. These interactions lead to platelet activation, increased pulmonary accumulation and modulation of platelet function. In turn, the ability of platelets to interact with immune and endothelial cells and secrete immune mediators modulate the outcome of lung infections and respiratory allergic diseases.

Associations of platelets with viral infections are just beginning to be identified. This highlights the complexity of platelet-mediated immune responses in infectious diseases (Figure 1).

## 5. Systemic Biomarkers of Platelet Activation with Predictive Potential

Upon activation, platelets express P-selectin and secrete their granule contents. Several markers of platelet activation such as P-selectin, CD40L, and PF4 have been identified to correlate with inflammation [112,130]. Also, platelet-monocyte complexes have been identified as markers for platelet activation in atherosclerosis [131]. These complexes have longer persistence in peripheral blood and are more sensitive markers of in vivo platelet activation. 

Platelet activation markers can be studied by enzyme-linked immunosorbent assay (ELISA), multiplex assay or flow cytometry using specific antibodies. Flow cytometry is currently the best standardized method to study platelet function, since measurements are independent of platelet counts and it allows both the analysis of the expression of platelet activation markers as well as the quantitation of platelet complexes with other blood cells [130,131,132,133]. Additionally, platelet micro-particles have more recently emerged as biological markers associated with platelet activation. Previous studies have reported that the levels of circulating platelet micro-particles were increased in patients with hypertension or atherosclerosis [134]. This could also be the case for infectious or allergic lung diseases.

Although important efforts in standardizing analytical variables have been developed, the application of efficient platelet collection methods, more accurate quantification technologies and a universal consensus of measurement standardization will help to advance the platelet field. Additional studies measuring systemic biomarkers of platelet activation on hospital admission will likely strengthen our knowledge on linking alterations in circulating platelet counts or platelet biomarkers to clinical indices of disease outcome and mortality.

## 6. Considerations in Human Platelet Research

Plateletcrit is the volume percentage occupied by platelets in blood and represents the total platelet mass. Plateletcrit is the result of multiplying MPV by platelet counts. Both parameters are easily obtained in a routine blood count. Normal plateletcrit is around 0.2% blood volume. It is remarkable that platelet functionality is concentrated in such a small volume. This fact raises the importance of platelet research in the context of health and disease.

**Figure 2:**
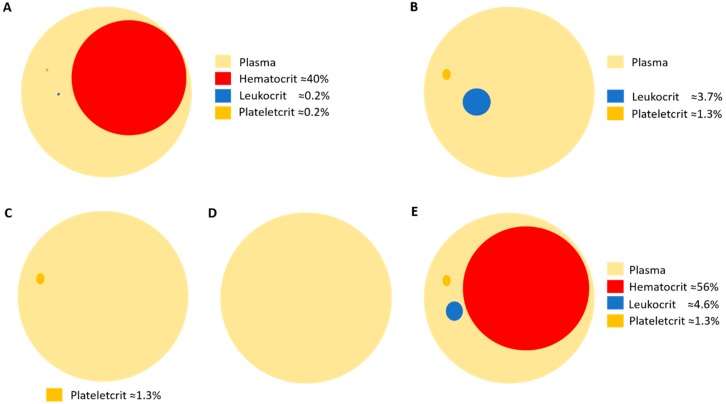
Schematic representation of the volume percentages of blood cell compartments in (**A**) whole blood; (**B**) buffy coat obtained after density-gradient centrifugation; (**C**) platelet-rich plasma (PRP) obtained by plateletpheresis; (**D**) plasma; and (**E**) leukoreduction system chamber (LRSC), a side-product of plateletpheresis.

Platelets have a mean density of 1.058 g/mL and a mean volume of 9 × 10^−15^ L [135]. As general research has traditionally focused on blood leukocytes, density-gradient media such as Lymphoprep™ or Ficoll-Paque^®^ have been broadly used for the isolation of these cell types. Ficoll-Paque^®^ has a density of 1.078 g/mL. This makes platelets lay between leukocytes and acellular plasma in the cell layer known as buffy coat after blood centrifugation. Thus, buffy coat contains most of the leukocytes and platelets from blood. This highlights two facts: 1) the properties attributed to leukocytes could be biased by platelet contamination, and 2) the extraction of pure platelet samples is difficult using classical laboratory techniques. Nevertheless, pure platelet collection is well standardized in blood transfusion centers that routinely process blood donations. Efficient platelet collection is usually performed by apheresis [136,137]. The main advantage of plateletpheresis collection is the purity of samples, containing no red cells or leukocytes. Plateletpheresis is not widely used for research purposes; however, this technique has opened the possibility to study platelet population without contamination with other blood constituents. Moreover, plateletpheresis allows collecting acellular plasma, in addition to platelet-rich plasma (PRP) samples. During the apheresis process, leukoreduction system chambers (LRSCs) are side-products of plateletpheresis usually discarded that represent an enriched alternative source of functional leukocytes [138]. Therefore, the apheresis procedure allows obtaining paired samples of leukocytes, platelets and plasma from patients, simplifying sampling and minimizing sample contamination. A recent study has been published using this technique [139] (Figure 2).

Besides platelet sampling, it is important to consider the standardization of platelet measurements by individual platelet counts. Currently, the lack of a common consensus in platelet determinations makes it difficult to generate universal conclusions [140,141]. This standardization process is key to make proper conclusions and, more importantly, it permits comparisons among different subjects, experiments and studies.

## 7. Conclusions

Emerging evidence suggests that platelets can no longer be considered as just key players in hemostasis and coagulation, but as major contributors to immune-mediated responses. Their ability to interact with immune cells as well as their load in immune mediators comprising both soluble molecules and membrane receptors makes them one attractive target for biomarker identification that will be useful in the treatment of infectious and allergic lung diseases. There are still long avenues to explore in the field of platelet immunity. We are just beginning to understand their immune role in both health and disease.

## Figures and Tables

**Table 1:** Summary of major platelet-derived inflammatory mediators and membrane receptors.

**α-Granules**		
**No.**	**Molecule**	**Function**
1	PF4 (CXCL4)	Chemokine: Induce leukocyte pro-inflammatory cytokine release in monocyte, neutrophil, and T-cell recruitment; Th differentiation
2	P-selectin	Adhesion molecule: Formation of platelet-leukocyte aggregate; Formation of bridges between leukocytes and endothelium
3	CD40L	TNF superfamily: antigen-presenting cell activation, B-cell responses, endothelial cell activation
4	MIP-1a (CCL3)	Cytokine: neutrophil and eosinophil activation, B-cell immunoglobulin production
5	IL-1β	Cytokine: acute phase response, leukocyte and endothelial activation
6	RANTES (CCL5)	Chemokine: Promotes monocyte, macrophage and T cell recruitment
7	TGF-β	Cytokine: cell proliferation, T-cell differentiation, B-cell and macrophage phenotype regulation
8	PDGF	Growth factor: cell growth and differentiation, monocyte/macrophage differentiation
9	VWF	Platelet adhesion, PMN extravasation
10	CD63	Tetraspanin: transmembrane adaptor protein, leukocyte recruitment
11	SDF-1	Chemokine: T-cell, monocyte, and PMN chemotaxis
12	VEGF	Growth factor: angiogenesis, adhesion molecule expression
13	Ppbp β-thromboglobulin (NAP-2)	Chemokine: neutrophil activation and recruitment, macrophage phagocytic activity
14	Thrombospondins	Apoptosis, endothelial cell inflammation, macrophage-platelet aggregates
15	MMP-2, MMP-9	Protease: extracellular matrix breakdown, platelet-leukocyte aggregate formation
16	Cyclophilin A	Vascular smooth muscle cell growth factor
18	CXCL1, CXCL5, CXCL7, CXCL12	Chemokines
19	Microbial proteins	Cationic proteins: disrupt cell membrane
**Dense Granule**		
**No.**	**Molecule**	**Immune/Inflammatory Role**
1	Serotonin	DC and T-cell functions
2	Glutamate	T-cell trafficking
3	Polyphosphates	Inflammatory response amplification
4	ADP	Platelet, leukocyte, endothelial cell activation
5	Histamine	Increased vessel reactivity and degranulation
6	ATP, phosphate, calcium	Fuel cell and co-factors in thrombosis
7	Eicosanoids	Pro-inflammatory signals
**Produced Metabolites**		
**No.**	**Molecule**	**Immune/Inflammatory Role**
1	Thromboxane	Eicosanoid: T-cell differentiation, monocyte activation
2	Nitric oxide	Reactive oxygen species: anti-inflammatory and antithrombotic
3	GPIbα	Adhesion molecule: binds Mac-1 on leukocytes
4	TXA2	Mediator that enhance platelet activation
5	S1P	Active metabolite which activate platelets and stimulate mitogenesis
6	PAF	Bioactive lipid: induce endothelial migration
7	Chrondroitin sulfate	Metabolite released by platelets after trigger complement activation
8	LPA	Lipid: ligand of G protein-coupled receptors
**Membrane Receptors**		
**No.**	**Molecule**	**Immune/Inflammatory Role**
1	TLR1, TLR2, TLR4, TLR6, TRL8 and TLR9	Receptors that recognize pathogen-associated molecular patterns and mediate inflammatory events
2	CD40, CD40L	Receptor: Mediator of interactions between lymphocytes and antigen presenting cells
3	GPIa, GPIIb/IIIa, GPIc-IIa (VLA-6)	Platelet glycoprotein: adhesion molecules
4	GPVI	Collagen receptor: induces powerful platelet activation
5	P2X1	Receptor is involved in platelet shape change and in activation by collagen
6	P2Y1, P2Y12	G-protein receptors: sustain platelet activation in response to ADP
7	PAR-1, PAR-4	Thrombin activates platelets through proteolytic cleavage of PAR receptors
8	ICAM-2,	Adhesion molecule
10	JAM-A,	Protects from thrombosis by suppressing integrin αIIbβ3

NOTE: ADP, adenosine 5′-diphosphate; CD40L, CD40 ligand; DC, dendritic cell; GPIba, glycoprotein Iba; 5-HT, 5-hydroxytryptamin; IL, interleukin; LPA, lysophosphatydic acid; MIP, macrophage-inflammatory protein; MMP, metalloproteinase; NAP, neutrophil-activating peptide; PAFR, platelet-activating factor receptor; PAR, protease-activated receptors; PDGF, platelet-derived growth factor; PF4, platelet factor 4; PMN, neutrophil; ppbp, proplatelet basic protein; SDF, stromal cell–derived factor; SP1, sphingosine-1-phosphate; TGF, transforming growth factor; Th, T helper; TLR, toll-like receptor; TNF, tumor necrosis factor; TxA2, Thromboxane A2; VEGF, vascular endothelial growth factor; VWF, von Willebrand factor.
